# Adipose Co-expression networks across Finns and Mexicans identify novel triglyceride-associated genes

**DOI:** 10.1186/1755-8794-5-61

**Published:** 2012-12-06

**Authors:** Blake E Haas, Steve Horvath, Kirsi H Pietiläinen, Rita M Cantor, Elina Nikkola, Daphna Weissglas-Volkov, Aila Rissanen, Mete Civelek, Ivette Cruz-Bautista, Laura Riba, Johanna Kuusisto, Jaakko Kaprio, Teresa Tusie-Luna, Markku Laakso, Carlos A Aguilar-Salinas, Päivi Pajukanta

**Affiliations:** 1 Department of Human Genetics and David Geffen School of Medicine at UCLA, Gonda Center, Room 6335B, 695 Charles E. Young Drive South, Los Angeles, California, 90095-7088, USA; 2 Department of Biostatistics, David Geffen School of Medicine at the University of California, Los Angeles, USA; 3 Obesity Research Unit, Department of Medicine, Division of Endocrinology, Helsinki University Central Hospital, and University of Helsinki, Helsinki, Finland; 4 Institute for Molecular Medicine FIMM, University of Helsinki, Helsinki, Finland; 5 Obesity Research Unit, Department of Psychiatry, Helsinki University Central Hospital, Helsinki, Finland; 6 Department of Medicine, David Geffen School of Medicine at the University of California, Los Angeles, USA; 7 Department of Endocrinology and Metabolism, Instituto Nacional de Ciencias Médicas y Nutrición Salvador Zubiran, Mexico City, Mexico; 8 Molecular Biology and Genomic Medicine Unit, Instituto Nacional de Ciencias Médicas y Nutrición, Salvador Zubiran, and Instituto de Investigaciones Biomédicas de la UNAM, Mexico City, Mexico; 9 Department of Medicine, University of Eastern Finland and Kuopio University Hospital, Kuopio, Finland; 10 Department of Mental Health and Substance Abuse Services, National Institute for Health and Welfare, Helsinki, Finland; 11 Hjelt Institute, Department of Public Health, University of Helsinki, Helsinki, Finland

**Keywords:** Mexicans, Finns, RNA sequencing, Triglycerides, Adipose tissue, Weighted gene co-expression network analysis

## Abstract

**Background:**

High serum triglyceride (TG) levels is an established risk factor for coronary heart disease (CHD). Fat is stored in the form of TGs in human adipose tissue. We hypothesized that gene co-expression networks in human adipose tissue may be correlated with serum TG levels and help reveal novel genes involved in TG regulation.

**Methods:**

Gene co-expression networks were constructed from two Finnish and one Mexican study sample using the blockwiseModules R function in Weighted Gene Co-expression Network Analysis (WGCNA). Overlap between TG-associated networks from each of the three study samples were calculated using a Fisher’s Exact test. Gene ontology was used to determine known pathways enriched in each TG-associated network.

**Results:**

We measured gene expression in adipose samples from two Finnish and one Mexican study sample. In each study sample, we observed a gene co-expression network that was significantly associated with serum TG levels. The TG modules observed in Finns and Mexicans significantly overlapped and shared 34 genes. Seven of the 34 genes (*ARHGAP30, CCR1, CXCL16, FERMT3, HCST, RNASET2, SELPG*) were identified as the key hub genes of all three TG modules. Furthermore, two of the 34 genes (*ARHGAP9, LST1*) reside in previous TG GWAS regions, suggesting them as the regional candidates underlying the GWAS signals.

**Conclusions:**

This study presents a novel adipose gene co-expression network with 34 genes significantly correlated with serum TG across populations.

## Background

Coronary heart disease (CHD) is the most common cause of death in the Western world. Serum triglyceride (TG) levels have been implicated as a well known risk factor for CHD [1]. Genome-wide association studies (GWAS) have been successful in identifying common variants and candidate genes influencing serum TG levels. A recent metaGWAS found, however, that the sum of their observed variants only explains 9.6% of the variation in TG levels [2], leaving a large portion of predicted genetic heritability of TGs (35%-48%) [3] undiscovered. Additionally, GWAS often have association signals spanning many genes, which results in several potential gene candidates that cannot be narrowed down using GWAS alone. The Mexican population has a high risk of dyslipidemia, with 31.5% of the population suffering from hypertriglyceridemia (TGs>1.7 mmol/l) [4]. Additionally, no genome-wide transcriptome analysis for lipids to date has been performed in Mexicans. Thus, exploring adipose tissue gene co-expression networks in Mexicans is necessary to better understand lipid regulation in this underinvestigated population. The Finnish population represents a genetically relatively homogeneous population which may help reduce genetic heterogeneity in the statistical analysis [5]. Adipose tissue is a key player in CHD progression through influencing systemic inflammation, insulin resistance, and serum TG levels [6]. In this study, we investigated whether serum fasting TG levels are related to adipose co-expression networks in human. Although synthesis of TGs occurs not only in adipose tissue but also in other tissues such as liver, hydrolysis of TGs, lipolysis, occurs predominantly in adipose tissue [7]. During fasting, adipose tissue hydrolyzes stored TGs and releases glycerol and fatty acids into the bloodstream. Previous studies have shown that excess TG hydrolysis in adipose tissue is associated with insulin resistance and can cause ectopic TG storage and high serum fatty acid levels [7]. Another relevant tissue for lipid metabolism is liver. However, obtaining liver biopsies from living study subjects for research purposes is complicated by the risks related to the procedure, making the adipose tissue a more accessible tissue than liver in humans. Previous studies have analyzed gene expression data from subcutaneous adipose tissue to search for genes involved in one of adipose tissue’s several functions [8-10]. However, to the best of our knowledge, no prior study has focused on discovering underlying lipid co-expression networks in adipose tissue shared across multiple populations. Weighted Gene Co-expression Network Analysis (WGCNA) is a well-established tool that identifies novel gene co-expression networks and searches for associations between a network and a phenotypic trait [10-14]. Determining novel genes and adipose networks correlated with serum TG levels consistently across populations helps lay the foundation for future functional studies to uncover the underlying mechanisms. We hypothesized that searching for TG-associated adipose networks preserved across populations may reveal novel genes involved in TG regulation. Comparing TG-associated gene expression networks from WGCNA in three independent sets of samples from adipose tissue of two different populations is a unique approach to discovering novel TG genes. The fact that the genes we discovered were members of TG modules in all three independent sets of samples and replicated across two populations provides strong evidence that the TG genes identified are genuine signals of biological importance.

## Results

### Weighted gene co-expression network analysis (WGCNA)

**Figure 1:**
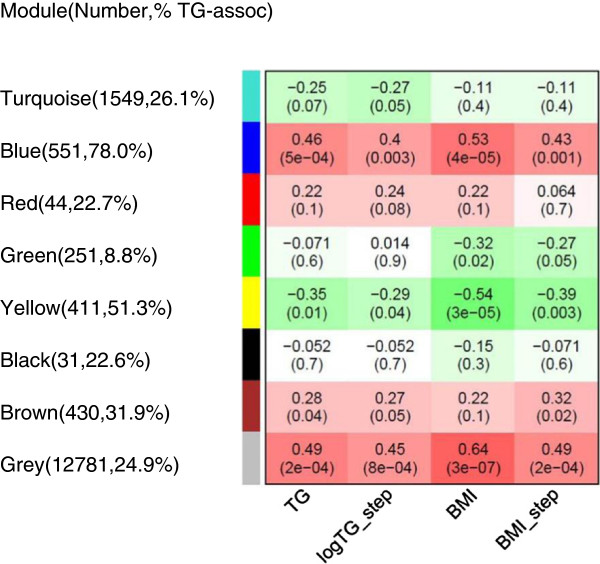
**Finnish twin WGCNA results indicate that the blue module is associated with serum TG levels.** Each row represents a module (labeled by color), and each column represents a trait. The value at the top of each square represents the correlation coefficient between the module eigengene and the trait with the correlation p-value in parentheses. The red color represents a positive correlation between the module eigengene and trait, and the green color represents a negative correlation. The number of gene expression probes present in each module and the percent of probes correlated with TGs (p<0.05, Pearson correlation) are listed in parenthesis next to the module color. To prevent potential confounding factors influencing expression values, gene expression values were corrected for age, sex, and twin relatedness using the Microarray Quality Control Pipeline as described previously [10]. TG values were log transformed and corrected for age, sex, disconcordant BMI status, and BMI using stepwise linear regression. BMI values were corrected for age, sex, disconcordant BMI status, and TG using stepwise linear regression. Genes that could not be clustered into one of the modules were assigned to the grey module, thus grey denotes background genes outside of modules. The blue module associated with TGs passed the Bonferroni correction for the 16 statistical tests performed (8 modules, 2 traits); the Bonferroni p-value cut-off = 3.1 x 10^-3^.

To search for novel gene co-expression networks involved in adipose TG regulation, we performed a gene co-expression network analysis using WGCNA [11,12] in 53 adipose RNAs from Finnish twins. The clinical characteristics of the twin study sample, including mean lipid values are shown in Additional file 1. To correct for the relatedness of the monozygotic twins, gene expression values were corrected for kinship (see methods). WGCNA identifies gene co-expression networks (modules), summarizing the main patterns of variation. Modules can be related to traits and sequence variants. The first principal component represents the summary of the module and is referred to as the module eigengene (ME) [11,12]. ME is the average module expression value for an individual. Relating MEs instead of genes to a clinical trait is a major advantage of WGCNA as it diminishes the multiple testing from thousands of transcripts to the number of modules. Thus, instead of testing correlation between every transcript and a trait, we only test the correlation between the ME value and the trait. We used two key metabolic traits to search for module-trait correlations in human adipose tissue: serum TGs and BMI. The two traits were tested as quantitative traits (by adjusting and not adjusting for covariates, see methods). We observed seven modules in the Finnish twins of which the blue module was positively correlated with serum TGs (*P*=5x10^-4^, Figure 1), indicating that TGs are higher in individuals with high ME values. The adjusted TG levels provided similar results (Figure 1). Figure 1 shows that the most significant TG modules were also implicated for BMI. It is worth noting that in WGCNA the grey module always represents background genes outside of modules, i.e. genes that cannot be clustered into one of the modules are assigned to the grey module. Thus, the grey module may contain genes that are associated with TGs but are not part of a WGCNA module.

### WGCNA TG modules preserved across populations

**Figure 2:**
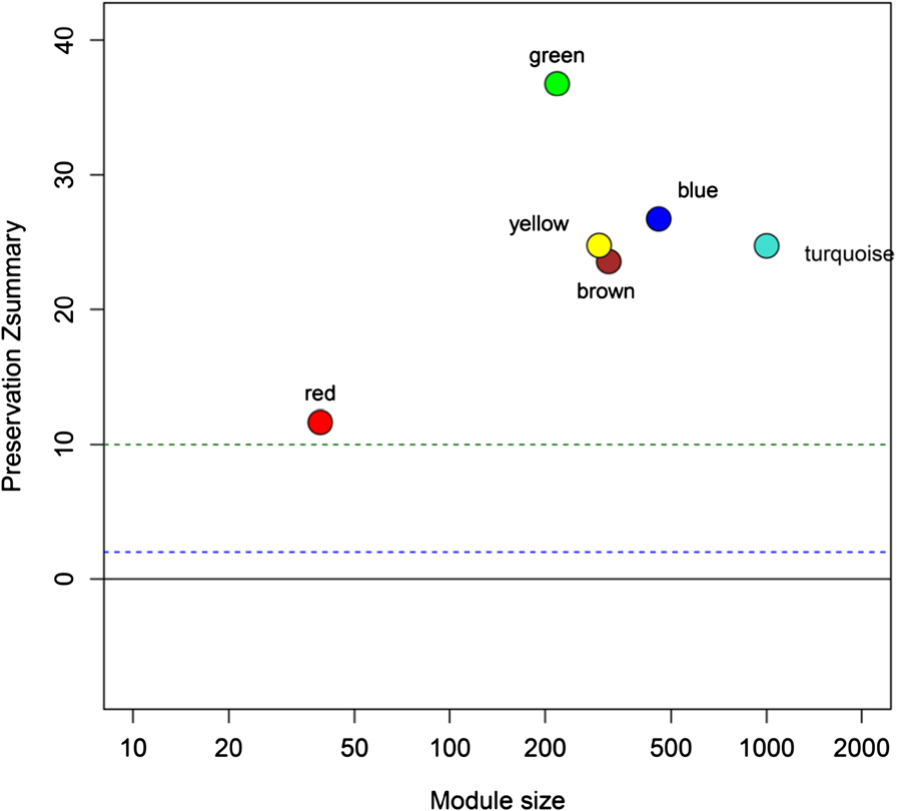
**Finnish twin WGCNA blue TG module is preserved in Mexican TG cases/controls.** Each data point represents a Finnish twin WGCNA module. A Zsummary value over 2 represents a moderately preserved module. A Zsummary value over 10 provides strong evidence of module preservation. To prevent potential confounding factors influencing expression values, Finnish twin gene expression values were corrected for age, sex, and twin relatedness using the Microarray Quality Control Pipeline as described previously [10].

**Figure 3:**
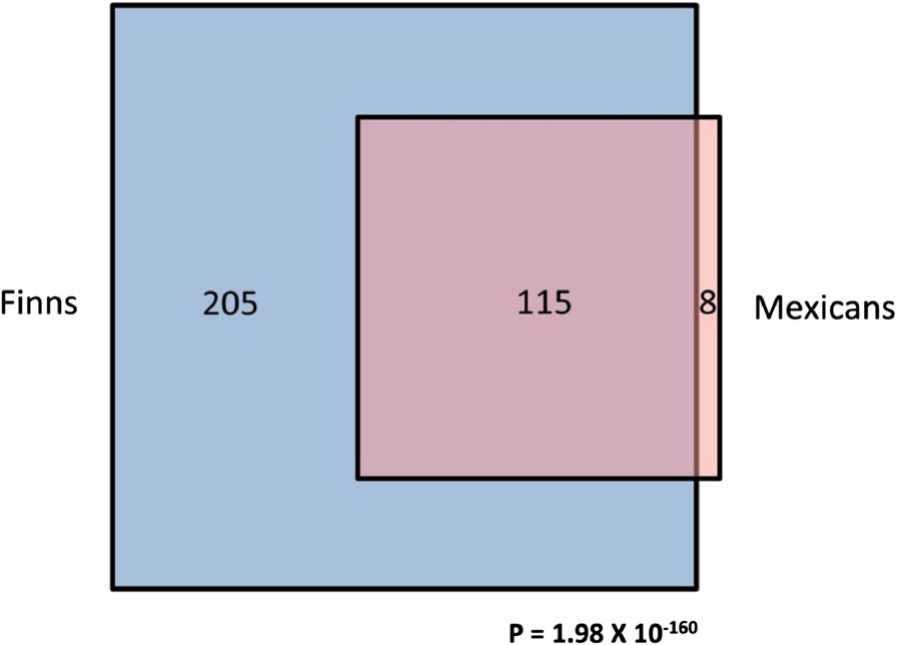
**Genes in the Finnish and Mexican TG modules significantly overlap.** The blue square represents Finnish TG module genes (n=320), and the pink square represents Mexican TG modules genes (n=123) determined by WGCNA. Area represented is proportional to the number of genes present in each group. P-value was calculated using a Fisher’s Exact test.

To test whether the TG-associated co-expression networks observed in a European origin population can be replicated in another population, we investigated whether the Finnish twin TG module is preserved in 70 Mexican TG case/control fat biopsy samples [10]. The clinical characteristics of the Mexican study sample, including mean lipid values are shown in Additional file 1. Mexican population represents an admixed population of European, Amerindian and African origin [15]. Figure 2 illustrates that the Finnish twin TG module is highly preserved in the Mexicans (Z score ~ 30). A preservation Z score >10 is considered significant preservation (see methods). Furthermore, the entire structure of adipose gene expression networks observed in the Finns was preserved in Mexicans, as all Finnish network modules had Z scores>10 (Figure 2). Given the observed significant module preservation between the Finns and Mexicans, we hypothesized that many of the genes detected in the Finnish TG network module could be replicated in Mexicans. In the WGCNA analysis of the Mexican TG case/control samples, we found eight modules of which the yellow module was significantly associated with serum TGs (*P*=1.0 x 10^-4^, Additional file 2). It is worth noting that as the color labels for WGCNA are given based on the number of probes found in the given module, module colors in different sets of samples are not comparable and thus, comparing the color labels observed between the sets of samples does not indicate whether the modules are related. Importantly, 115 of the 123 genes in the yellow Mexican TG module were shared between the Finnish and Mexican TG modules, and thus 93.5% of the TG module genes in Mexicans overlapped with the Finns (*P*=1.98 x 10^-160^ for the overlap, Figure 3). We also investigated the module membership of the genes shared between the Mexican and Finnish TG modules. The module membership of a gene is defined as the correlation coefficient of its expression profile and the corresponding module eigengene across samples (see methods). We observed that the module memberships of the genes shared between the Mexican and Finnish TG modules are highly correlated (r=0.67, p=1.56x10^-16^), providing additional evidence that the relevant TG genes and network modules are strongly conserved between the Mexican and Finnish sets of samples, because in both populations the genes have similar degrees of relatedness to the TG module.

To search for enrichments in biological gene ontology (GO) functions linking the Finnish and Mexican TG modules to adipose TG regulation, we utilized the DAVID database [16,17]. We observed five significantly enriched (Bonferroni corrected *P*<0.001) gene ontology (GO) biological process terms present in both the Finnish and Mexican modules, all including immunity and inflammation categories (Table 1). Taken together, these data provide novel evidence that the gene expression signatures in human adipose tissue are correlated with serum TG levels in a highly similar way across different populations. To further establish the robustness of the gene sets found in the Finnish and Mexican TG modules, we performed WGCNA in a third sample, adipose RNAs from 20 Finnish METSIM TG cases/controls that were analyzed using a different gene expression approach, RNAseq. The clinical characteristics of the METSIM study sample, including mean lipid values are shown in Additional file 1. This comparison of the Affymetrix microarray and RNAseq data should decrease the well-known variability and errors in the measurement methods of gene expression. We observed that the brown module in the METSIM TG case/control sample was significantly associated with TG levels (*P*=2.0 x 10^-4^, Additional file 3). Furthermore, the Finnish twin TG module was the most preserved module in the METSIM TG case/controls (Additional file 4). Importantly, 34 of the 115 genes shared between the Finnish twins and Mexicans overlapped with the Finnish METSIM TG module (*P*<10^-10^ for the overlap). We focused our subsequent studies on these 34 genes as they represent the significant overlap robustly observed across populations and different gene expression approaches. It is also worth noting that the TG trait provided a significantly associated network module passing Bonferroni correction for the two tested traits and the number of modules in all three sets of samples whereas no such consistent network was observed for BMI (Figure 1, Additional files 2, 3). Furthermore, when we looked into the percentages of genes correlated versus not correlated with TGs in all network modules throughout the three sets of samples, we observed that the TG-associated network modules in each of the three sets of samples (i.e. the blue module in the Finnish twins, the yellow module in the Mexicans and the brown module in the METSIM TG case/control sample) contain the highest percentages of genes correlated with TGs (Figure 1, Additional files 2, 3). All of the WGCNA network analyses identifying the 34 genes were performed using quantitative TG values. To investigate whether the across populations overlap was robust to the analytical approach, we also investigated TGs as a binary trait by classifying the subjects to high and low TG groups (see methods) in the conventional differential gene expression analysis. Using this qualitative TG approach, we discovered that the sets of samples also show a significant overlap in the genes that are differentially expressed between the high and low TG groups. Table 2 illustrates that the genes that are most differentially expressed between the TG extremes are shared between the sets of samples. Of the 37 genes common to all 3 top 500 differentially expressed gene lists, nine are also part of all three TG modules (*P*<10^-7^ for the overlap). Of the nine genes shared between these two lists, five lie within 500kb of a suggestive TG GWAS signal (p<10^-3^) [2], providing additional evidence that these genes are involved in TG regulation. The observation that the WGCNA TG module genes significantly overlap with conventional differential gene expression analysis provides additional evidence that the TG regulatory genes observed using the GO functional enrichment analysis in human adipose tissue are highly shared across Finns and Mexicans.

**Table 1:** Gene ontology categories enriched in both Mexican and Finnish twin TG modules

	**Finnish twins**		**Mexican**	
Gene Ontology Biological Process	Bonferroni	# probes	Bonferroni	# probes
Defense Response	3.06 × 10^-11^	46	7.82 × 10^-18^	34
Immune Response	2.42 × 10^-9^	46	1.82 × 10^-18^	36
Inflammatory Response	2.29 × 10^-5^	27	9.09 × 10^-10^	21
Response to Wounding	4.17 × 10^-5^	36	1.35 × 10^-8^	25
Positive Regulation of Immune Response	4.25 × 10^-2^	14	6.54 × 10^-4^	11

All p-values were corrected for multiple testing using the Bonferroni correction. The Gene Ontology biological process categories and the corresponding p-values were obtained using the GO_BP_FAT category of the DAVID bioinformatics database [16,17].

**Table 2:** The most differentially expressed genes using conventional gene expression analysis overlap between the three studies

		**p-values**	
Group	Top 100 Genes*	Top 250 Genes	Top 500 Genes
Finnish Twins, Mexicans	2.81 × 10^-33^	2.01 × 10^-69^	6.51 × 10^-103^
METSIM, Finnish Twins	3.27 × 10^-6^	5.96 × 10^-7^	5.22 × 10^-10^
METSIM, Mexicans	2.11 × 10^-4^	2.48 × 10^-5^	2.24 × 10^-8^

*Genes were sorted in descending order by the difference in the average normalized gene expression values between the high and low TG groups. The overlap p-values were calculated using a two-tail Fisher’s exact test. All p-values pass the Bonferroni correction for the nine statistical tests performed (p-value<5.6 x 10^-3^).

To search for a GO functional enrichment in which the 34 genes are involved, we utilized the DAVID database. The top four GO biological process categories observed between the Finnish twins and Mexicans (Table 1) were also significantly enriched among the 34 genes shared by all three sets of samples (Additional file 5). To identify hub genes that may be driving the three TG network modules, we searched for genes with high module membership (i.e. high correlation between gene expression and the module eigengene [the average module expression value for an individual]) in all three TG modules. We found seven genes (*ARHGAP30, CCR1, CXCL16, FERMT3, HCST, RNASET2, SELPG*) among the 34 shared genes that had significantly high module membership values in the top 50% in all three TG modules (*P*<10^-7^ for the overlap, Table 3), and that are thus driving the three TG modules. Two of these seven genes (*CCR1* and *CXCL16*) have previously been implicated in type 2 diabetes, CHD, or inflammation (Additional file 6). To determine whether any of the known genes of adipose TG regulation are correlated with the three TG modules, we investigated correlations of these positive control genes/gene families (*MGAT* family, *DGAT* family, and *LPL*), and observed that expression of all 4 *MGAT* genes tested (*MGAT1*, *MGAT2*, *MGAT4A*, *MGAT4B*) were significantly correlated (*P*<0.05) with TG module kME (i.e. the correlation between a gene expression value and the module eigengene [the average module expression value for an individual], see methods) in two or more of the sets of samples, although they were not placed into the module using WGCNA. It is worth noting that modules are formed based on similar gene expression patterns across multiple samples, so TG-associated genes that do not exhibit highly correlated gene expression will be found in different modules, and the grey module will contain genes associated with TGs not belonging to a WGCNA module. *MGAT2* and *MGAT4B* were also correlated (*P*<0.05) with serum TG values in at least two of the sets of samples. These data support the biological relevance of the new TG modules shared across three sets of samples and two populations.

**Table 3:** Genes with high module membership in the Finnish twins, Mexican, and METSIM triglyceride modules

**3 Triglyceride module overlap**	**Gene(s)**	**Empirical p-value**
1 gene with module membership* > 0.9	*CCR1*	6.18 × 10^-4^
2 genes in top 50 module membership	*HCST, FERMT3*	2.46 × 10^-6^
3 genes in top 25% module membership	*CCR1, CXCL16, HCST*	< 10^-7^
7 genes in top 50% module membership	*CCR1, CXCL16, HCST, ARHGAP30, FERMT3, RNASET2, SELPG*	< 10^-7^

*Module membership indicates the correlation coefficient between the module eigengene and gene expression.

The triglyceride modules referenced above are the brown METSIM module, the blue Finnish Twin module, and the yellow Mexican module. P-values were calculated empirically by randomly selecting the observed number of genes satisfying the module membership criteria and observing the number of overlaps occurring by chance.

### Investigation of common SNPs near TG module genes and TG GWAS signals

To determine whether any significant TG GWAS SNPs [2] may be acting through any of the 34 genes, we explored regions harboring significant TG GWAS variants around the 34 genes (±500 kb). We found that both *ARHGAP9* and *LST1* lie near a genome-wide significant TG GWAS signal^2^ (Additional files 7, 8), and seven other TG module genes shared between the 3 sets of samples (*CD52, CXCL16, HCST, MS4A7, NCKAP1L, PTPN6, and SPP1*) lie within 500kb of a suggestive TG GWAS signal (p<10^-3^) [2]. To determine whether the significant TG GWAS SNPs or SNPs in high LD with them in the *ARHGAP9* and *LST1* regions influence the observed TG modules, we tested the GWAS SNPs in the METSIM sample for correlation with the TG modules. We did not observe a significant correlation between rs11614506 in LD (r^2^=1) with the lead TG signal (rs11613352) in the *ARHGAP9* region (Additional file 7) and the METSIM TG case/control brown module. However, in the *LST1* region (Additional file 8), a SNP rs486416 that represents an independent signal from the lead TG GWAS signal, rs2247056, was significantly associated with the brown TG module (*P*=0.005), while the lead TG GWAS signal rs2247056 was not significantly associated with the brown TG module (*P*>0.05). Additionally, rs486416 is correlated with *LST1* expression (r=0.52, *P*=0.02), providing a possible mechanism underlying the SNP/TG module and TG module/serum TG level associations. SNP rs486416 exhibited a p-value of 1.99x10^-15^ for TGs in the previous metaGWAS [2], thus further suggesting *LST1* as one of the underlining genes for this region.

## Discussion

We investigated whether human adipose transcript networks are correlated with serum TG levels across populations to identify novel TG-associated genes for future targeted sequencing and functional studies. Using WGCNA, we discovered that there are gene co-expression networks in human adipose tissue significantly correlated with serum TGs and highly conserved across populations. The vast majority (93.5%) of the genes in the Mexican TG network module overlapped with the genes in the Finnish twin TG network module. Furthermore, the module membership of the genes shared between the Finnish and Mexican TG modules significantly correlate with each other (r=0.67, *P*=1.56x10^-16^). We also observed a significant overlap with 34 genes shared across the TG modules of all three sets of samples robust enough to be detected using two different gene expression approaches, microarrays and RNAseq. These 34 TG-associated genes identified in WGCNA also significantly overlap with the top 500 most differentially expressed genes in all three sets of samples (*P*<10^-7^ for the overlap), further validating the WGCNA approach. The gene co-expression network data of three independent sets of samples from Finnish and Mexican populations provide strong evidence that the 34 shared genes are involved in TG regulation in both Finns and Mexicans, and probably other populations as well. Furthermore, the fact that the TG associations remain when BMI is used as a covariate in stepwise regression provides evidence that the TG module associations are not due to differences in adiposity between the subjects. However, inferences about causation based on the WGCNA method must be supported by both biological arguments and statistically significant associations. We also recognize that some of the 34 genes may be reactive rather than causal. Taking into account these limitations, future targeted sequencing and functional studies are warranted.

The strength of module membership also significantly overlaps in all three TG modules. Seven of the 34 genes with high module membership were observed as hub genes, acting as key drivers of three TG modules (*P*<10^-7^ for the overlap, Table 3), spanning three sets of samples and two gene expression platforms. This finding implicates these seven genes in TG regulation, although additional experimentation is necessary to search for a specific molecular role they play in regulating TGs. Further studies are also warranted to determine whether the same 34 genes implicated are associated with TGs stored in adipose tissue, because adipose TG values were not available for our sets of samples. Two of the 34 genes (*ARHGAP9* and *LST1*) are located within 500 kb of a significant TG metaGWAS signal, while seven other genes lie near a suggestive TG GWAS signal [2]. Teslovich and coworkers, however, suggest different regional gene candidates associated with each of these two TG GWAS signals (i.e. *HLA-B* and *LRP1*) [2]. In the *LRP1* region, *ARHGAP9* identified in our study is located nearer the actual GWAS SNP than *LRP1*, and in the *HLA* region there were several non-redundant, significantly associated SNPs not in LD [2], one of which is located next to *LST1*, making *LST1* a likely second hit for the region.

Determining whether *ARHGAP9* or *LST1* expression quantitative trait loci (eQTLs) are driving the TG-associated WGCNA modules may reveal a novel mechanism of TG regulation. The recent metaGWAS study [2] reports that none of the lead TG-associated GWAS SNPs near HLA and *LRP1* were *cis*-eQTLs in adipose tissue. However, significant non-redundant regional SNPs that are not in LD with the lead SNP were not tested in this previous study [2]. Therefore, untested significant regional TG-associated GWAS SNPs may act as *cis*-eQTLs in adipose tissue, and they should thus be further explored. Our analysis demonstrate that a significant TG GWAS SNP rs486416 in the *HLA/LST1* region [2] is driving the adipose co-expression network associated with TGs in the METSIM TG case/control study sample by influencing *LST1* expression, thus providing a novel mechanism underlying the TG metaGWAS signal in the *HLA/LST1* region. The *ARHGAP9* region (±500kb) had only one significant TG GWAS signal [2] that was not significantly correlated with the brown TG module in METSIM TG cases/controls, in accordance with the previous cis-eQTL study [2]. We also discovered that 11 of the 34 genes found in all three TG modules show prior evidence of involvement in obesity, type 2 diabetes, or CHD (Additional file 6). The remaining 23 genes present novel TG candidates for future targeted sequencing and functional studies.

## Conclusions

Our cross-ethnic network analysis provides evidence that adipose gene co-expression networks correlated with serum TG levels are significantly conserved across two populations, the Mexicans and Finns. Within the WGCNA networks, 34 genes significantly overlapped and seven of them (*ARHGAP30, CCR1, CXCL16, FERMT3, HCST, RNASET2, SELPG*) displayed strong module membership in all three sets of samples, implicating them as the key candidates for TG regulation in human adipose tissue.

## Methods

The study design was approved by the ethics committees of the participating centers and all subjects gave a written informed consent.

### Sets of samples

#### Finnish twin cohort

A total of 53 Finnish individuals (26 monozygotic twin pairs concordant and discordant for BMI and one single co-twin) were recruited for surgical abdominal subcutaneous fat biopsies at the Helsinki University Hospital, Finland [18]. RNA was extracted and gene expression was measured using Affymetrix U133 Plus 2.0 gene chips, according to the manufacturer’s instructions and as described previously [18]. To ensure that each probe is expressed above background, gene expression probes that were expressed in at least half of the 53 samples were included in the analysis. To prevent potentially confounding factors influencing expression values, gene expression values were corrected for age, sex, and twin relatedness using the Microarray Quality Control Pipeline as described previously [10], resulting in 16,048 probes passing quality control. Lipid values were measured using enzymatic methods from Roche Diagnostic Hitachi tools, as described previously [18]. LogTG and BMI were corrected for significant covariates using stepwise regression. LogTG covariates tested included age, sex, disconcordant twin BMI status, and BMI. BMI covariates tested included age, sex, disconcordant twin BMI status, and TG.

#### Mexican TG cases/controls

Seventy Mexican high TG cases and low TG controls were recruited at the Instituto Nacional de Ciencias Medicas y Nutricion, Salvador Zubiran, Mexico City, Mexico for surgical abdominal subcutaneous fat biopsies [10]. RNA was purified from subcutaneous adipose tissue and the microarray data was normalized using the Microarray Quality Control Pipeline, as previously described [10], resulting in 14,942 probes passing quality control. TG values were measured using enzymatic methods (SERA-PAK), as previously described [19]. Gene expression values were corrected for age, sex, and kinship, and lipid values were measured as described previously [19]. LogTG and BMI were corrected for significant covariates using stepwise regression. LogTG covariates tested included age, sex, TG case/control status, and BMI. BMI covariates tested included age, sex, TG case/control status, and TG.

#### METSIM Cohort

The Finnish population-based cohort, METSIM (METabolic Syndrome In Men) was collected at the University of Kuopio, Kuopio, Finland as described previously [20]. The METSIM cohort is a randomly ascertained sample of 10,197 men aged 45–70 from Kuopio, Eastern Finland [20]. Twenty METSIM subjects with high TG (3.57 ± 1.69 mg/dl) or low TG (0.56 ± 0.06 mg/dl) values who were not on lipid lowering medication were selected for RNA sequencing. RNA was extracted from subcutaneous adipose tissue of the 20 METSIM subjects, and lipid measurements were performed using nuclear magnetic resonance (NMR), as described previously [21,22]. LogTG and BMI were corrected for significant covariates using stepwise regression. LogTG covariates tested included age, sex, TG case/control status, and BMI. BMI covariates tested included age, sex, TG case/control status, and TG.

### RNA Sequencing

We prepared the samples for RNA-sequencing using the Illumina mRNA-Sequencing kit, following the manufacturer’s instructions. Single-end, 50bp reads were produced using Illumina HiSeq2000 sequencing platform. Reads passing Illumina’s default quality control were aligned to the ENSEMBL v62 reference genome using the default options of TopHat v1.3.3 [23,24], a package for aligning RNAseq data to a reference genome. The expression level of a gene was determined with the Cufflinks software package [25-27] using the TopHat output to compute gene expression values as FPKM (Fragments per kilobase of exon per million mapped reads). FPKM is calculated by counting the number of reads spanning a gene and dividing by the length of the gene (in kilobases) and the number of reads in a lane that map to the genome (in millions). FPKM accounts for the variability in mapped reads seen across sequencing lanes and gene length to improve the accuracy of the gene expression value. On average, 100,225,314 reads mapped to 14,223 RefSeq genes after the QC was performed.

### Weighted gene co-expression network analysis (WGCNA)

We used WGCNA, which is a systems biology analysis method, to identify TG-associated co-expression modules and their key constituents [11,12,28]. WGCNA starts from the level of thousands of genes, identifies modules of co-expressed genes, and relates these modules to clinical variables and gene ontology information. Because gene modules may correspond to biological pathways, focusing the analysis on modules (and their highly connected intramodular hub genes) amounts to a biologically meaningful data reduction scheme. WGCNA is implemented in the R software package. Briefly, WGCNA uses a network distance coupled with hierarchical clustering and dynamic tree cutting to define modules as branches of a cluster tree. Modules (clusters) are defined in an unbiased fashion and initially denoted by colors. Grey denotes background genes outside of modules. The highly correlated module genes of a given module are represented and summarized by their first principal component (which is referred to as the module eigengene). The module eigengenes are used to define measures of module membership which quantify how close a gene is to a given module. Specifically, module membership (aka kME) is defined as the correlation between a gene expression value and the module eigengene (the average module expression value for an individual). Module membership measures allow one to annotate all genes on the array and to screen for disease related intramodular hub genes [29]. As described below, we use functional enrichment analysis with regard to known gene ontologies to understand the biological significance of module genes and to identify intramodular hub genes as candidate driver genes. WGCNA was applied separately to each of the three gene expression datasets: Finnish twins, Mexican TG case/control and METSIM. UCSC genes expressed in METSIM samples (n= 13,822 genes) were inputted into WGCNA, while all expressed probes in the Mexican samples (14,942 probes) and Finnish twin samples (16,048 probes) were used to form the WGCNA networks. We utilized the blockwiseModules R function of WGCNA [11,12] to determine co-expression networks formed in adipose tissue in each of the three populations. This approach has been used in several previous systems genetic applications, e.g. in Plaisier at al. [10]. The module colors are determined by the number of genes/probes within the module. Therefore, comparing the color labels observed between the 3 sets of samples does not indicate whether the modules are related (i.e. the yellow module in the Mexican study sample is not comparable to the yellow module in the Finnish Twin study sample). When evaluating the significance of the module correlations of WGCNA, we corrected the p-values for the number of modules and the number of tested phenotypic traits.

### Module preservation

Our module preservation analysis is based on a permutation test implemented in the modulePreservation R function [30]. The modulePreservation function implements a permutation test involving several powerful network based statistics for evaluating module preservation. These statistics are summarized into the composite preservation called Zsummary. For each module in the first data set (referred to as reference data), the function calculates the Zsummary statistic in the second data set (referred to as test data). For a given module, Zsummary>10 indicates strong evidence for preservation in the test data set. Zsummary < 2 indicates no evidence of module preservation. An advantage of the preservation Z statistic is that it makes few assumptions regarding module definition and module properties.

### Cross-tabulation based tests of module overlap

To merge expression data sets measured on different platforms, we used the collapseRows R function [29]. Briefly, this method first collapses multiple probes by gene symbol which allows one to merge the data by gene symbol [31]. The microarray probe with the greatest variance between samples was used to represent a given gene in the Mexican and Finnish twin samples.

To determine the relationship between two module assignments in two different data sets, we used standard cross-tabulation based methods (Fisher’s Exact test). We placed each expressed gene into a 2x2 categorical contingency table based on whether the gene was part of a TG-associated module in the two study samples tested. To determine the likelihood of overlapping modules, genes were randomly selected using the criteria specified in Table 3 and an empirical p-value was calculated after 10 million trials to accurately model the likelihood of these results occurring by chance. For the empirical p-value of the overlap between the three modules, we considered 10 billion trials sufficient as it would allow us to reach a p-value level of 10^-10^. We utilized the GO BP FAT function of DAVID to determine gene ontology biological processes enriched in the genes overlapping in all 3 TG modules [16,17]. Genes expressed in each dataset were used as the background measurement for DAVID.

### Gene expression overlap in TG cases and controls

In addition to the WGCNA analysis that was performed using quantitative TG values, we also performed differential gene expression analysis using a qualitative TG approach. In METSIM, Mexican, and Finnish twin sets of samples, each individual was labeled as a case or control using 1.5 mmol/L cut off, which represents the average fasting serum TG values for European origin male adults reported by the American Heart Association [32]. We used a two-tailed Fisher’s exact test to determine the significance of the TG module overlap. Six of 53 Finnish twins had TG values > 1.5 mmol/L, 10 of 20 METSIM samples had TG values > 1.5 mmol/L, and 44 of the Mexican TG case/controls had TG values > 1.5 mmol/L.

### Gene expression - TG correlations

In the three sets of samples we compared the percentages of genes that were correlated with TGs in each module. For these comparisons, correlation p-values were calculated using Pearson correlation between gene expression and quantitative TG values.

#### Additional file descriptions

The following additional data are available with the online version of this paper. Additional data file 1 is a table listing the clinical characteristics of the Finnish and Mexican sets of samples. Additional data file 2 is a figure of the WGCNA results in the Mexican study sample. Additional file 3 is a figure of the WGCNA results in the METSIM study sample. Additional file 4 is the module preservation results for the Finnish twin and Mexican sets of samples. Additional file 5 is a table of the significant DAVID gene enrichment results. Additional file 6 is a table describing the relevance of the TG module overlap genes to CHD-related traits. Additional file 7 is a figure illustrating the GWAS results in the ARHGAP9 region from a prior TG GWAS. Additional file 8 is a figure illustrating the GWAS results in the LST1 region from a prior TG GWAS. In the three sets of samples we compared the percentages of genes that were correlated with TGs in each module. For these comparisons, correlation p-values were calculated using Pearson correlation between gene expression and quantitative TG values.

## Abbreviations

*ARHGAP30*: Rho GTPase activating protein 30; BMI: Body mass index; *CCR1*: Chemokine (C-C motif) receptor 1; CHD: Coronary heart disease; *CXCL16*: Chemokine (C-X-C motif) ligand 16; eQTL: Expression quantitative trait locus; *FERMT3*: Fermitin family homolog 3; FPKM: Fragments per kilobase of exon per million mapped reads; GO: Gene ontology; GWAS: Genome-wide association study; *HCST*: Hematopoietic cell signal transducer; HDL-C: High density lipoprotein cholesterol; *HLA*: Human leukocyte antigen; *HLA-B*: Human leukocyte antigen B; LD: Linkage disequilibrium; *LRP1*: Lipoprotein receptor-related protein 1; NMR: Nuclear magnetic resonance; *PLTP*: Phospholipid transfer protein; ME: Module eigengene; METSIM: Metabolic Syndrome in Men; *RNASET2*: Ribonuclease T2 precursor; *SELPG*: Selectin P ligand; TC: Total cholesterol; TG: Triglycerides; WGCNA: Weighted Gene Co-expression Network Analysis.

## Competing interests

The authors declare that they have no competing interests.

## Authors' contributions

TT-L, CAA-S, IC-B, and LR collected the Mexican study sample and performed the microarray measurements. SH, RMC, DW-V, and MC participated in the data analysis. EN performed the RNA sequencing. JKu and ML collected the METSIM sets of samples and measured microarray gene expression. KHP, JKa, and AR collected the Finnish twin sets of samples and performed microarray experiments. BEH participated in the experimental design, analyzed the data, and wrote the manuscript. PP developed the experimental design, participated in the data analyses, and wrote the manuscript. All authors read and/or helped write the manuscript, and approved of the manuscript.

## Pre-publication history

The pre-publication history for this paper can be accessed here:

http://www.biomedcentral.com/1755-8794/5/61/prepub

## Supplementary Material

Additional file 1 **Clinical characteristics of the Finnish and Mexican sets of samples.** Additional data file 1 is a table listing the clinical characteristics of the Finnish and Mexican sets of samples. Click here for file

Additional file 2 **The WGCNA results show that the yellow module is associated with serum TG levels in the Mexican TG cases/controls.** Additional data file 2 is a figure of the WGCNA results in the Mexican sets of samples. Click here for file

Additional file 3 **The WGCNA results indicate that the brown module is associated with serum TG levels in the METSIM TG cases/controls.** Additional file 3 is a figure of the WGCNA results in the METSIM sets of samples. Click here for file

Additional file 4 **The Finnish Twin WGCNA blue TG module is moderately preserved in the METSIM TG case/control study sample.** Additional file 4 is the module preservation results for the Finnish twin and Mexican sets of samples. Click here for file

Additional file 5 **The 34 genes found in the Finnish twin, METSIM, and Mexican TG modules are enriched for immunity and inflammation genes.** Additional file 5 is a table of the significant DAVID gene enrichment results. Click here for file

Additional file 6 **List of the 34 genes that overlap in all three WGCNA triglyceride modules in the Finnish Twins, METSIM, and Mexican sets of samples.** Additional file 6 is a table describing the relevance of the TG module overlap genes to CHD-related traits. Click here for file

Additional file 7 **The TG metaGWAS results in the ARHGAP9 region (+/− 500kb) utilizing the publicly available data from Teslovich et al. 2012.** Additional file 7 is a figure illustrating the GWAS results in the ARHGAP9 region from a prior TG GWAS. Click here for file

Additional file 8 **The TG metaGWAS results in the LST1 region (+/− 500kb) utilizing the publicly available data from Teslovich et al. 2010.** Additional file 8 is a figure illustrating the GWAS results in the LST1 region from a prior TG GWAS. Click here for file
