# Supplementary material for: Adipose Co-expression networks across Finns and Mexicans identify novel triglyceride-associated genes
Source: BMC Med Genomics. 2012 Dec 6;5:61. doi: 10.1186/1755-8794-5-61 (PMC3543280; doi:10.1186/1755-8794-5-61)
Supplement: Additional file 1 — Clinical characteristics of the Finnish and Mexican sets of samples. Additional data file 1 is a table listing the clinical characteristics of the Finnish and Mexican sets of samples. [file 1755-8794-5-61-S1.pdf]

Additional File 1. Clinical characteristics of the Finnish and Mexican study samples.

Trait	Mexican TG		Finnish Twin	METSIM TG	
	Cases	Controls		Cases	Controls
n (male)	44(28)	25(7)	53 (25)	10 (10)	10 (10)
Age (years)	37.8 ± 9.2	37.8 ± 9.3	28.9 ± 4.2	56.3 ± 4.1	51.9 ± 2.6
TG (mmol/L)	4.5 ± 2.4	1.1 ± 0.3	1.1 ± 0.4	3.6 ± 1.7	0.6 ± 0.1
BMI (kg/m^2)	26.7 ± 2.3	24.5 ± 2.9	27.7 ± 4.2	29.4 ± 2.7	25.0 ± 3.3
Total Chol (mmol/L)	6.4 ±1.3	4.7 ± 0.9	4.4 ± 0.7	6.5 ± 0.7	5.0 ± 0.6
HDL (mmol/L)	1.1 ± 0.2	1.3 ± 0.3	1.4 ± 0.4	1.1 ± 0.2	2.1 ± 0.7
Fasting Glucose (mmol/L)	5.5 ± 1.3	5.0 ± 0.5	5.3 ± 0.5	6.0 ± 0.7	5.6 ± 0.7
Fasting Insulin (mmol/L)	13.8 ± 9.1	8.9 ± 6.0	6.5 ± 3.8	11.3 ± 3.2	4.5 ± 1.9

Trait values are displayed as mean ± standard deviation.
