# Supplementary material for: Adipose Co-expression networks across Finns and Mexicans identify novel triglyceride-associated genes
Source: BMC Med Genomics. 2012 Dec 6;5:61. doi: 10.1186/1755-8794-5-61 (PMC3543280; doi:10.1186/1755-8794-5-61)
Supplement: Additional file 3 — The WGCNA results indicate that the brown module is associated with serum TG levels in the METSIM TG cases/controls. Additional file 3 is a figure of the WGCNA results in the METSIM sets of samples. [file 1755-8794-5-61-S3.pdf]

Additional file 3. The WGCNA results indicate that the brown module is associated with serum TG levels in the METSIM TG cases/controls.

Module(Number, % TG-assoc)

Red(502, 2.0%)
Magenta(300, 19.0%)
Yellow(1030, 2.6%)
Black(370, 8.1%)
Brown(1444, 79.9%)
Pink(315, 1.3%)
Turquoise(5415, 22.6%)
LightYellow(84, 11.9%)
RoyalBlue(80, 2.5%)
Purple(220, 2.3%)
MidnightBlue(109, 0.9%)
Cyan(111, 0.0%)
Grey60(100, 2.0%)
Salmon(128, 0.0%)
LightCyan(104, 0.0%)
Tan(186, 0.0%)
Blue(2166, 3.0%)
GreenYellow(193, 1.0%)
Green(835, 5.0%)
LightGreen(95, 2.1%)
Grey(35, 5.7%)

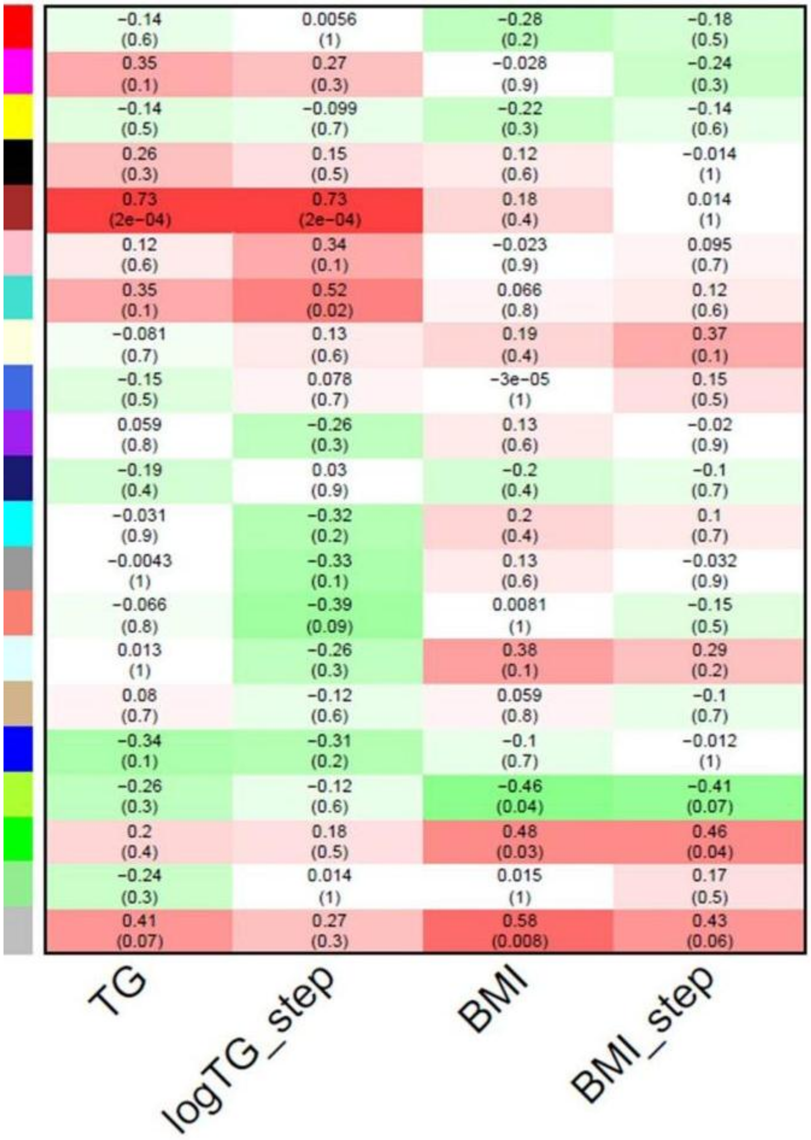

Each row represents a module (labeled by color), and each column represents a trait. The value at the top of each square represents the correlation coefficient between the module eigengene and the trait. Beneath the correlation coefficient in parentheses is the correlation p-value. The red color represents

a positive correlation between the module eigengene and the trait, and the green color represents a negative correlation. The number of genes present in each module and the percent of genes correlated with TGs ($p<0.05$, Pearson correlation) are listed in parenthesis next to the module color. Genes that cannot be clustered into one of the modules are assigned to the Grey module. TG values were log transformed and corrected for age, sex, TG status, and BMI using stepwise linear regression. BMI values were corrected for age, sex, TG status, and TG using stepwise linear regression. As the color labels for WGCNA are given based on the number of probes found in the given module, module colors in different study samples are not comparable and thus, comparing the color labels observed between the study samples does not indicate whether the modules are related. The brown module-TG association passed the Bonferroni multiple testing correction for the 42 statistical tests performed (21 modules, 2 traits); the Bonferroni p-value cut-off = 1.2×10^{-3}.
