# Supplementary material for: Adipose Co-expression networks across Finns and Mexicans identify novel triglyceride-associated genes
Source: BMC Med Genomics. 2012 Dec 6;5:61. doi: 10.1186/1755-8794-5-61 (PMC3543280; doi:10.1186/1755-8794-5-61)
Supplement: Additional file 4 — The Finnish Twin WGCNA blue TG module is moderately preserved in the METSIM TG case/control study sample. Additional file 4 is the module preservation results for the Finnish twin and Mexican sets of samples. [file 1755-8794-5-61-S4.pdf]

Additional file 4. The Finnish Twin WGCNA blue TG module is moderately preserved in the METSIM TG case/control study sample.

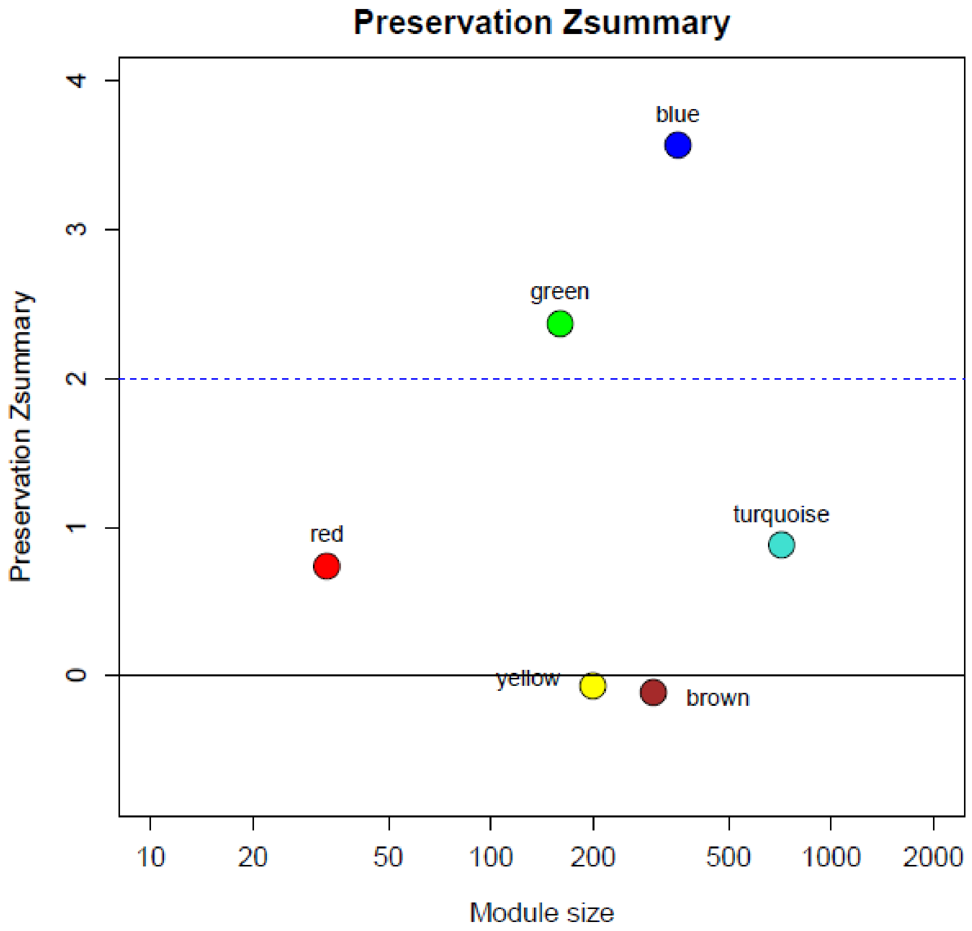

Each data point represents a Finnish twin WGCNA module. A Zsummary value over 2 represents a moderately preserved module[1,2]. To prevent potential confounding factors influencing expression values, Finnish twin gene expression values were corrected for age, sex, and twin relatedness using the Microarray Quality Control Pipeline as described previously[3].

Additional file 4 references

1. Langfelder P, Zhang B, Horvath S. **Defining clusters from a hierarchical cluster tree: the Dynamic Tree Cut package for R.** *Bioinformatics* 2008, **24:**719-720.
2. Langfelder P, Horvath S. **WGCNA: an R package for weighted correlation network analysis.** *BMC Bioinformatics* 2008, **9:**559.
3. Plaisier CL, Horvath S, Huertas-Vazquez A, Cruz-Bautista I, Herrera MF Tusie-Luna T, Aguilar-Salinas C, Pajukanta P: **A systems genetics approach implicates USF1, FADS3, and other causal candidate genes for familial combined hyperlipidemia.** *PLoS Genet* 2009, **5:**e1000642.
