# Supplementary material for: Adipose Co-expression networks across Finns and Mexicans identify novel triglyceride-associated genes
Source: BMC Med Genomics. 2012 Dec 6;5:61. doi: 10.1186/1755-8794-5-61 (PMC3543280; doi:10.1186/1755-8794-5-61)
Supplement: Additional file 5 — The 34 genes found in the Finnish twin, METSIM, and Mexican TG modules are enriched for immunity and inflammation genes. Additional file 5 is a table of the significant DAVID gene enrichment results. [file 1755-8794-5-61-S5.pdf]

Additional file 5. The 34 genes found in the Finnish twin, METSIM, and Mexican TG modules are enriched for immunity and inflammation genes.

Gene Ontology Biological Process	Genes (Known/Total)	Bonferroni P-value
Defense Response	6/10	3.46×10^{-5}
Immune Response	3/9	1.38×10^{-3}
Inflammatory Response	5/7	4.58×10^{-3}
Response to Wounding	6/8	1.12×10^{-2}

All p-values were corrected for multiple testing using the Bonferroni correction. The Gene Ontology biological process categories and the corresponding p-values were obtained using the "GO_BP_FAT" category of the DAVID bioinformatics database. Known indicates prior evidence of a potential involvement in obesity, type 2 diabetes, or coronary heart disease. Total indicates the number of 34 genes that are in the GO biological process.
