# Supplementary material for: Adipose Co-expression networks across Finns and Mexicans identify novel triglyceride-associated genes
Source: BMC Med Genomics. 2012 Dec 6;5:61. doi: 10.1186/1755-8794-5-61 (PMC3543280; doi:10.1186/1755-8794-5-61)
Supplement: Additional file 6 — List of the 34 genes that overlap in all three WGCNA triglyceride modules in the Finnish Twins, METSIM, and Mexican sets of samples. Additional file 6 is a table describing the relevance of the TG module overlap genes to CHD-related traits. [file 1755-8794-5-61-S6.pdf]

Additional file 6. List of the 34 genes that overlap in all three WGCNA triglyceride modules in the Finnish Twins, METSIM, and Mexican study samples.

Gene Symbol	Gene Name	lipid, obesity, and CHD Information
ALOX5AP	arachidonate 5-lipoxygenase-activating protein	myocardial infarction and stroke pathogenesis[1], increased subcutaneous adipose expression is associated with obesity & type II diabetes[2]
ARHGAP30	Rho GTPase activating protein 30	
ARHGAP9	Rho GTPase activating protein 9	
C1orf38	basement membrane-induced gene	
C3AR1	complement component 3a receptor 1	overexpressed in adipose tissue of obese subjects[3]
CCR1	chemokine (C-C motif) receptor 1	increased subcutaneous adipose tissue expression in obese subjects[4]
CD163	cluster of differentiation 163	increased subcutaneous adipose tissue expression in obese subjects, correlated with macrophage abundance[5]
CD48	cluster of differentiation 48	
CD52	cluster of differentiation 52	
CXCL16	chemokine (C-X-C motif) ligand 16	Increases LDL uptake in aortic smooth muscle; atherosclerosis progression[6]
CYBB	Cytochrome b beta chain	overexpressed in subcutaneous adipose tissue of obese subjects[3]
EVI2B	ecotropic viral integration site 2B precursor	

Gene Symbol	Gene Name	lipid, obesity, and CHD Information
FCER1G	Fc fragment of IgE, high affinity I, receptor	
FERMT3	fermitin family homolog 3	
GLIPR1	glioma pathogenesis-related 1 precursor	
HCLS1	hematopoietic cell-specific Lyn substrate 1	
HCST	hematopoietic cell signal transducer	
IL10RA	Interleukin 10 receptor, alpha variant	upregulated in subcutaneous adipose tissue during evoked inflammation[7]
LCP2	lymphocyte cytosolic protein 2	
LST1	leukocyte specific transcript 1	
LYN	Yamaguchi sarcoma viral (v-yes-1) oncogene	
MS4A7	membrane-spanning 4-domains, subfamily A, member	
NCF2	neutrophil cytosolic factor 2	
NCKAP1L	NCK-associated protein 1-like	

Gene Symbol	Gene Name	lipid, obesity, and CHD Information
PTPN6	protein tyrosine phosphatase, non-receptor type	upregulated in omental adipose tissue of obese individuals[8]
PTPRJ	protein tyrosine phosphatase, receptor type, J	
RAC2	ras-related C3 botulinum toxin substrate 2	upregulated in subcutaneous adipose tissue of obese individuals on saturated fatty acid diet[9]
RGS10	regulator of G-protein signaling 10	
RNASET2	ribonuclease T2 precursor	
SELPLG	selectin P ligand	snps associated with coronary heart disease[10]
SLCO2B1	solute carrier organic anion transporter family,	
SPP1	secreted phosphoprotein 1	plasma and adipose SPP1 levels increased in obese individuals[11]
SRGN	serglycin precursor	
VSIG4	V-set and immunoglobulin domain containing 4	

The triglyceride modules referenced above are the brown METSIM module, the blue Finnish Twin module, and the yellow Mexican module.

Additional file 6 References

1. Helgadottir A, Manolescu A, Thorleifsson G, Gretarsdottir S, Jonsdottir H, Thorsteinsdottir U, Samani NJ, Gudmundsson G, Grant SF, Thorgeirsson G, Sveinbjornsdottir S, Valdimarsson EM, Matthiasson SE, Johannsson H, Gudmundsdottir O, Gurney ME, Sainz J, Thorhallsdottir M, Andresdottir M, Frigge ML, Topol EJ, Kong A, Gudnason V, Hakonarson H, Gulcher JR, Stefansson K: **The gene encoding 5-lipoxygenase activating protein confers risk of myocardial infarction and stroke.** *Nat Genet* 2004, **36:**233-239.
2. Kaaman M, Rydén M, Axelsson T, Nordström E, Sicard A, Bouloumié A, Langin D, Arner P, Dahlman I: **ALOX5AP expression, but not gene haplotypes, is associated with obesity and insulin resistance.** *Int J Obes (Lond)* 2006, **30:**447-452.
3. Dahlman I, Kaaman M, Olsson T, Tan GD, Bickerton AS, Wåhlén K, Andersson J, Nordström EA, Blomqvist L, Sjögren A, Forsgren M, Attersand A, Arner P: **A unique role of monocyte chemoattaractant protein 1 among chemokines in adipose tissue of obese subjects.** *J Clin Endocrinol Metab* 2005, **90:**5834-5840.
4. Huber J, Kiefer FW, Zeyda M, Ludvik B, Silberhumer GR, Prager G, Zlabinger GJ, Stulnig TM: **CC chemokine and CC chemokine receptor profiles in visceral and subcutaneous adipose tissue are altered in human obesity.** *J Clin Endocrinol Metab* 2008, **93:**3215-3221.
5. Shakeri-Manesch S, Zeyda M, Huber J, Ludvik B, Prager G, Stulnig TM: **Diminished upregulation of visceral adipose heme oxygenase-1 correlates with waist-to-hip ratio and insulin resistance.** *Int J Obes (Lond)* 2009, **33:**1257-1264.

6. Chandrasekar B, Bysani S, Mummidi S: **CXCL16 signals via Gi, phosphatidylinositol 3-kinase, Akt, I kappa B kinase, and nuclear factor-kappa B and induces cell-cell adhesion and aortic smooth muscle cell proliferation.** *J. Biol Chem* 2004, **279:**3188-3196.
7. Shah R, Lu Y, Hinkle CC, McGillicuddy FC, Kim R, Hannenhalli S, Cappola TP, Heffron S, Wang X, Mehta NN, Putt M, Reilly MP: **Gene profiling of human adipose tissue during evoked inflammation in vivo.** *Diabetes* 2009, **58:**2211-2219.
8. Gómez-Ambrosi J, Catalán V, Diez-Caballero A, Martinez-Cruz LA, Gil MJ, García-Foncillas J, Cienfuegos JA, Salvador J, Mato JM, Frühbeck G: **Gene expression profile of omental adipose tissue in human obesity.** *FASEB J* 2004, **18:**215-217.
9. van Dijk SJ, Feskens EJ, Bos MB, Hoelen DW, Heijligenberg R, Bromhaar MG, de Groot LC, de Vries JH, Müller M, Afman LA: **A saturated fatty acid-rich diet induces an obesity-linked proinflammatory gene expression profile in adipose tissue of subjects at risk of metabolic syndrome.** *Am J Clin Nutr* 2009, **90:**1656-1664.
10. Bugert P, Vosberg M, Entelmann M, Jahn J, Katus HA, Klüter H: **Polymorphisms in the P-selectin (CD62P) and P-selectin glycoprotein ligand-1 (PSGL-1) genes and coronary heart disease.** *Clin Chem Lab Med* 2004, **42:**997-1004.
11. Gómez-Ambrosi J, Catalán V, Ramírez B, Rodríguez A, Colina I, Silva C, Rotellar F, Mugueta C, Gil MJ, Cienfuegos JA, Salvador J, Frühbeck G: **Plasma osteopontin levels and expression in adipose tissue are increased in obesity.** *J Clin Endocrinol Metab* 2007, **92:**3719-3727.
