# Supplementary material for: Adipose Co-expression networks across Finns and Mexicans identify novel triglyceride-associated genes
Source: BMC Med Genomics. 2012 Dec 6;5:61. doi: 10.1186/1755-8794-5-61 (PMC3543280; doi:10.1186/1755-8794-5-61)
Supplement: Additional file 7 — The TG metaGWAS results in the ARHGAP9 region (+/− 500kb) utilizing the publicly available data from Teslovich et al. 2012. Additional file 7 is a figure illustrating the GWAS results in the ARHGAP9 region from a prior TG GWAS. [file 1755-8794-5-61-S7.pdf]

Additional file 7. The TG metaGWAS results in the ARHGAP9 region (+/- 500kb) utilizing the publicly available data from Teslovich et al. 2010[1].

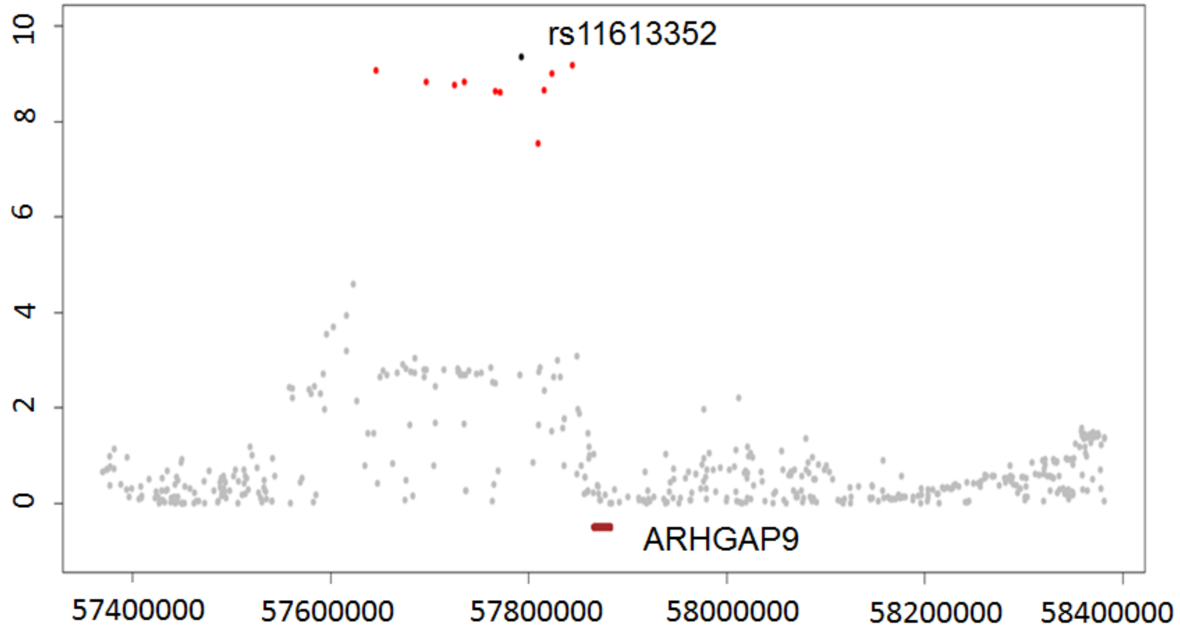

The black datapoint represents the strongest TG GWAS signal from Teslovich et al.[1] in this region, and the red datapoints represent SNPs in LD ($r^2 > 0.50$) with the primary signal. Grey datapoints depict SNPs that are not in LD ($r^2 < 0.50$) with the primary GWAS signal. ARHGAP9 is indicated in brown. The Y-axis represents the –log(p) TG GWAS value from Teslovich et al.[1], and the X-axis the basepair position on human chromosome 12.

ARHGAP9 is located 74 kb from a GWAS signal associated with TGs and HDL (rs11613352)[1]. The gene predicted to be causal in this region is the low density lipoprotein receptor-related protein 1 (*LRP1*), which is 186 kb away from the implicated SNP rs11613352. When including SNPs in LD with the key SNP (r^2>0.5), the physical distance between the TG GWAS signal and *LRP1* decreases to 39 kb, and the distance between the TG GWAS signal and *ARHGAP9* decreases to 22 kb. Although *LRP1* appears as a likely regional candidate given its

known involvement in lipid reglation, *ARHGAP9* remains a very plausible candidate as well because the gene is found in all 3 TG WGCNA modules and represents the nearest gene to the GWAS signal.

Additional file 7 references:

1. Teslovich TM, Musunuru K, Smith AV, Edmondson AC, Stylianou IM, Koseki M, Pirruccello JP, Ripatti S, Chasman DI, Willer CJ, Johansen CT, Fouchier SW, Isaacs A, Peloso GM, Barbalic M, Ricketts SL, Bis JC, Aulchenko YS, Thorleifsson G, Feitosa MF, Chambers J, Orho-Melander M, Melander O, Johnson T, Li X, Guo X, Li M, Shin Cho Y, Jin Go M, Jin Kim Y et al.: **Biological, clinical and population relevance of 95 loci for blood lipids.** *Nature* 2010, **466:**707-713.
