# Supplementary material for: Adipose Co-expression networks across Finns and Mexicans identify novel triglyceride-associated genes
Source: BMC Med Genomics. 2012 Dec 6;5:61. doi: 10.1186/1755-8794-5-61 (PMC3543280; doi:10.1186/1755-8794-5-61)
Supplement: Additional file 8 — The TG metaGWAS results in the LST1 region (+/− 500kb) utilizing the publicly available data from Teslovich et al. 2010. Additional file 8 is a figure illustrating the GWAS results in the LST1 region from a prior TG GWAS. [file 1755-8794-5-61-S8.pdf]

Additional file 8. The TG metaGWAS results in the LST1 region (+/- 500kb) utilizing the publicly available data from Teslovich et al. 2010[1].

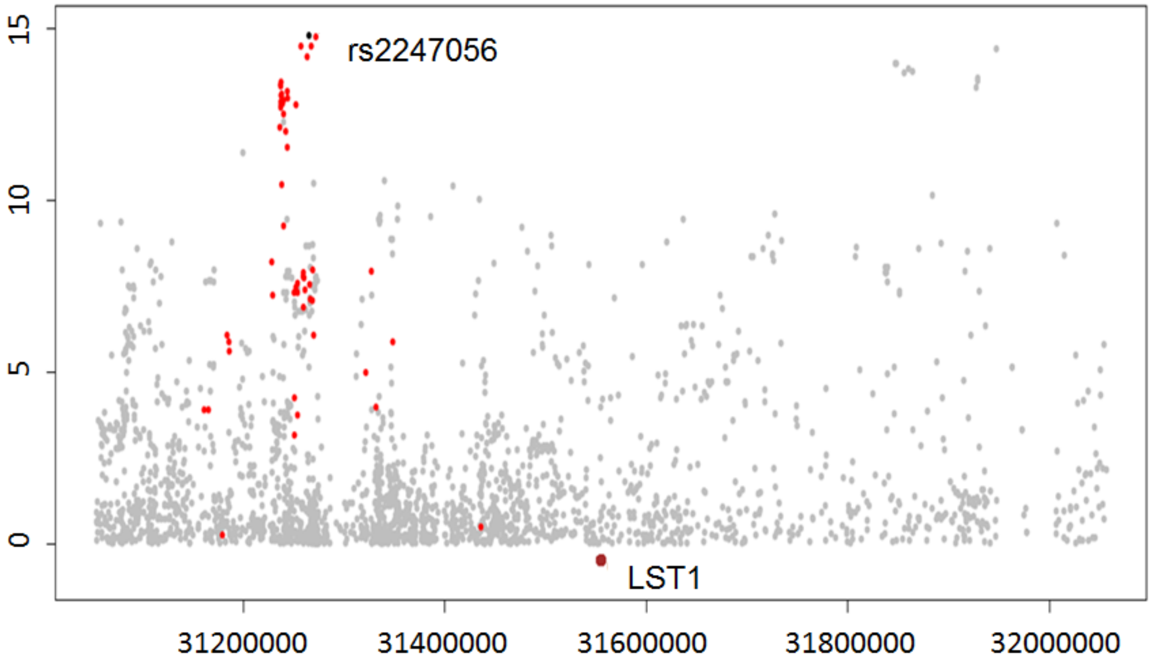

The black datapoint represents the strongest TG GWAS signal from Teslovich et al.[1] in this region, and the red datapoints represent SNPs in LD ($r^2 > 0.50$) with the primary signal. Grey datapoints indicate SNPs that are not in LD ($r^2 < 0.50$) with the primary GWAS signal. LST1 is depicted in brown. The Y-axis represents the –log(p) TG GWAS value from Teslovich et al.[1], and the X-axis the basepair position on human chromosome 6.

LST1 is located 289 kb away from a SNP significantly associated with TGs in the metaGWAS (rs2247056)[1]. The SNP rs2247056 is an intronic SNP residing within *HLA-B*. Howver, there are other independent SNPs tha pass the GWAS p-value cut-off of $5x10^{-8}$ not in LD with rs2247056 ($r^2<0.5$). In fact, one SNP (rs1800629) within 11 kb of *LST1* is significantly associated with TGs (P=7.9 X 10^{-9}). Thus, *LST1* remains a plausible candidate for this region,

as it is found in all 3 TG WGCNA modules and the region harbors multiple independent TG signals.

Additional file 8 references:

1. Teslovich TM, Musunuru K, Smith AV, Edmondson AC, Stylianou IM, Koseki M, Pirruccello JP, Ripatti S, Chasman DI, Willer CJ, Johansen CT, Fouchier SW, Isaacs A, Peloso GM, Barbalic M, Ricketts SL, Bis JC, Aulchenko YS, Thorleifsson G, Feitosa MF, Chambers J, Orho-Melander M, Melander O, Johnson T, Li X, Guo X, Li M, Shin Cho Y, Jin Go M, Jin Kim Y et al.: **Biological, clinical and population relevance of 95 loci for blood lipids.** *Nature* 2010, **466:**707-713.
